# Hypervirulent *Clostridium difficile* PCR-Ribotypes Exhibit Resistance to Widely Used Disinfectants

**DOI:** 10.1371/journal.pone.0025754

**Published:** 2011-10-25

**Authors:** Lisa F. Dawson, Esmeralda Valiente, Elizabeth H. Donahue, George Birchenough, Brendan W. Wren

**Affiliations:** Department of Infectious and Tropical Diseases, London School of Hygiene and Tropical Medicine, London, United Kingdom; Loyola University Medical Center, United States of America

## Abstract

The increased prevalence of *Clostridium difficile* infection (CDI) has coincided with enhanced transmissibility and severity of disease, which is often linked to two distinct clonal lineages designated PCR-ribotype 027 and 017 responsible for CDI outbreaks in the USA, Europe and Asia. We assessed sporulation and susceptibility of three PCR-ribotypes; 012, 017 and 027 to four classes of disinfectants; chlorine releasing agents (CRAs), peroxygens, quaternary ammonium compounds (QAC) and biguanides. The 017 PCR-ribotype, showed the highest sporulation frequency under these test conditions. The oxidizing biocides and CRAs were the most efficacious in decontamination of *C. difficile* vegetative cells and spores, the efficacy of the CRAs were concentration dependent irrespective of PCR-ribotype. However, there were differences observed in the susceptibility of the PCR-ribotypes, independent of the concentrations tested for Virkon®, Newgenn®, Proceine 40® and Hibiscrub®. Whereas, for Steri7® and Biocleanse® the difference observed between the disinfectants were dependent on both PCR-ribotype and concentration. The oxidizing agent Perasafe® was consistently efficacious across all three PCR ribotypes at varying concentrations; with a consistent five Log10 reduction in spore titre. The PCR-ribotype and concentration dependent differences in the efficacy of the disinfectants in this study indicate that disinfectant choice is a factor for llimiting the survival and transmission of *C. difficile* spores in healthcare settings.

## Introduction

*Clostridium difficile-*infection (CDI) is an antibiotic associated diarrhoea, caused by *C. difficile*, a Gram-positive, spore-forming anaerobic bacillus. CDI clinical symptoms can range from mild diarrhoea to life threatening pseudomembranous colitis. Antibiotic therapy is proposed to elicit CDI by disruption of the intestinal microbiota, which enables colonization of the gastrointestinal tract by indigenous or ingested *C. difficile*. *C. difficile* was first recognized as a pathogen over 30 years ago, and primarily CDI was associated with immune suppressed and elderly patients, receiving antibiotic treatment [1]. However, in the last 10 years *C. difficile* has emerged as a global pathogen, with epidemics across Europe, Asia and the USA, culminating in the transcontinental spread of ‘hypervirulent’ PCR-ribotypes [2], [3], [4]. Evolutionary and genetic analysis of *C. difficile* have revealed five distinct clonal lineages, Clades 1–5 inclusive, which are conserved across analysis methods such as microarray [5], MLST sequence type (ST) [6] and whole genome sequencing [7]. The most notable being the PCR-ribotype 027/Clade 1/ST-1 and 017/Clade 4/ST-37, which have brought a concomitant increase in disease severity, mortality, recurrence rate, enhanced relative transmissibility and decreased mean age of infection [4], [8], [9]. Consequently, *C. difficile* is the most frequent cause of nosocomial diarrhoea worldwide [10], [11]. *C. difficile* has a unique advantage over other healthcare associated communicable infections such as methicillin resistant *Staphlococcus aureus* (MRSA), due to its ability to form spores, which are central to transmission of *C. difficile.* Patients with *C. difficile* are estimated to excrete between 1×10^4^ and 1×10^7^ spores per gram of faeces [12], [13]. Spores are highly infectious and readily transmissible [13], hence they are particularly problematic in healthcare settings [14], as they are able to persist on a variety of surfaces [15], [16], [17], [18] and are resistant to many disinfectants [19], [20], [21]. The use of disinfectants in combating the spread of CDI in hospitals and the community is central to infection control strategies, particularly as studies indicate a correlation between overlapping resistance mechanisms to disinfectants, antiseptics and antibiotics [22], [23]. Adaptation to altered antibiotic treatment regimes has been met with modified antimicrobial resistance patterns within *C. difficile* isolates [24], [25], [26], which is particularly apparent within the 027 lineage, whereby some 027 isolates have acquired fluoroquinolone resistance [7].

Resistance to antibiotics and disinfectants is a potential problem in managing infection control. There is a broad selection of disinfectants available, with differing active compounds. Presently, the UK Department of Health and Health Protection Agency guidelines advocate the use of chlorine-based disinfectants at a concentration of 1000 ppm for disinfection of *C. difficile*.

Representative isolates of the 012, 017 and 027 PCR-ribotypes were chosen for analysis; strain 630 is an 012 PCR ribotype is a virulent multidrug resistant strain isolated from an outbreak in a Zurich hospital in 1982 [27], and was therefore isolates before may of the disinfectants in this study were manufactured. Strain 630 was the first *C. difficile* genome to be fully sequenced [28]. Strain R20291 is a representative 027 PCR-ribotype that was isolated from an outbreak in Stoke Mandeville hospital in 2006 and strain M68 is a representative 017 PCR-ribotype that was isolated from a CDI outbreak in Ireland in 2006, both of which have been fully sequenced [7]. We tested the susceptibility of spores and vegetative cells from the 012, 017 and 027 PCR-ribotypes to a panel of nine commercially available biocides from four categories of disinfectant. These include chlorine releasing agents (CRAs), peroxygen releasing agents, quaternary ammonium compounds (QACs), and a chlorhexidine based hand wash.

With the exception of Perasafe®, the disinfectants fell into three categories, i) those whose efficacy were concentration dependent, independent of the PCR-ribotype, ii) those whose efficacy were PCR-ribotype dependent and iii) those whose efficacies were dependent on both PCR-ribotype and concentration. Perasafe® was the only disinfectant consistently efficacious across all three PCR ribotypes at varying concentrations, where survival was below the limit of detection.

## Results

### Sporulation of *C. difficile* PCR-ribotypes

**Figure 1:**
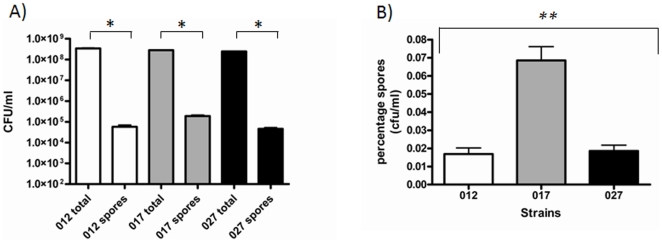
Vegetative cells and Spore counts of *C. difficile* PCR ribotypes 012, 017 and 027. A) Total cell counts and spore counts were obtained by plating cultures and heat resistant samples of *C. difficile* on blood plates containing 0.1% taurocholate. B) Percentage spore counts were obtained by calculating the number of heat resistant spores as a proportion of the total cell counts. Data consists of three biological and two technical replicates from separate cultures. Student T-tests were performed between total counts and spores for each strain and significant differences are marked with a bracket * (*p*<0.05). A comparison for percentage survival of spores was performed using linear regression and a partial F-test, where M68 was the reference strain, a significant difference (p<0.01) in spore production between the three strains is marked with a bracket **.

Spore production is a unique feature of *C. difficile* among other important healthcare pathogens, therefore vegetative cell production and sporulation of three representative PCR-ribotypes 012, 017 and 027 (Figure 1a) was analysed. The 012 and 027 strains exhibited similar levels of sporulation in minimal media, 5.7×10^4^ CFU/ml and 5.1×10^4^ CFU/ml respectively, whereas the 017 strain spore titre was significantly higher, 1.8×10^5^ (*p<*0.0000 Partial F-test) (Figure 1b). This observation was consistent with heat resistant spores and microscopy counted spores (Spores were counted using a Neubauer-ruled Bright Line counting chambers; Hausser Scientific data not shown).

### Susceptibility to disinfectants

The susceptibility of 012, 017 and 027 PCR ribotypes to a panel of disinfectants was assessed *in-vitro* using pure *C. difficile* cultures at 2.9×10^8^ (±0.5). Preliminary investigations were performed with contact times of 2 minutes, 30 minutes and 4 hours. There were no significant differences between the data obtained at these time points, therefore a 30 minute contact was used throughout for experimental ease. The disinfectants used in the study are listed in Table 1. The data is expressed on a Log plot as normalized CFU/ml to take into account the differences in spore production between the three ribotypes. Statistical analysis (see methods) was performed to address three questions i) is there a strain dependent sensitivity to the disinfectants? ii) if so, what is the most appropriate concentration to use? and iii) which disinfectant has the greatest efficacy across all three PCR-ribotypes and concentrations?

**Table 1:** The disinfectants used in this study.

Disinfectant name	Biocide type	Active ingredient(s)	Recommended concentration	Recommended uses	Manufacturer
Actichlor®	CRA	sodium dichloroisocyanurate	1000 ppm (5000 ppm*)	blood and body fluid spills	Ecolab
		(Troclosene Sodium)		and for general hygiene	
Bioclense®	QAC	Benzalkonium chloride	5%	surfaces and general hygiene	Teknon
HazTab®	CRA	Sodium Dichloroisocyanurate	1000 ppm (10000 ppm*)	blood and body fluid spills	Guest Medical LTD
		(Sodium dichloro-1,3,5 triazinetrione dihydrate)		and for general hygiene	
Hibiscrub®	Cationic bis-biguanide	chlorhexidine gluconate	100%	Handwash	Regent Medical
NewGenn®	QAC	Di-decyl dimethyl ammonium chloride	0.8%	surfaces, general hygiene and equipment	NewwGenn research
PeraSafe®	Peroxygen	peracetic acid	1.62%	medical devices, surfaces and general hygiene	Micro Medical
Proceine 40®	QAC	alkyl-amino-alkyl glycines	0.6%	small spills, surfaces and general hygiene	AGMA
Steri 7®	QAC	Isothiazolium-benzalkonium chloride	100%	general hygiene and surfaces	Sentinal International LTD
Virkon®	Peroxygen	potassium peroxymonosulfate	1%	hazardous spills, surfaces and equipment	DuPont

The active ingredients, biocide type and recommended working concentrations and recommended uses are listed. Outbreak or blood spill concentrations are highlighted with * where they differ from the standard working concentrations. There were no minimum contact times provided for the disinfectants.

### Chlorine releasing agents

**Figure 2:**
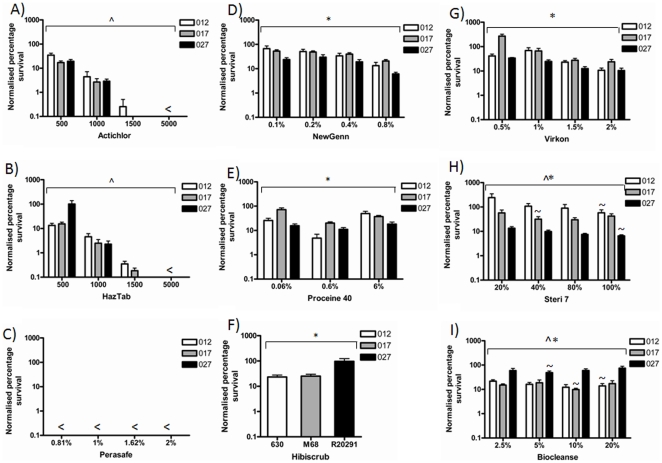
Exposure of *C. difficile* PCR-ribotype 012, 017 and 027 strains to disinfectants. Percentage survival after 30 minute exposure to A) Actichlor® at 5000 ppm, 1500 ppm, 1000 ppm and 500 ppm. B) Haztab® at 5000 ppm, 1500 ppm, 1000 ppm and 500 ppm. C) Perasafe® at 2%, 1.62%, 1% and 0.81%. D) Virkon® at 2%, 1.5%, 1% and 0.5%. E) Biocleanse® at 20%, 10%, 5% and 2.5%. F) Newgenn® at 0.8%, 0.4%, 0.2% and 0.1%. G) Proceine 40® at 6%, 0.6% and 0.06%. H) Steri 7® at 100%, 80%, 40% and 20%. I) Hibiscrub® at 50%. The survival was calculated as a percentage of the heat resistant spore counts from unchallenged cultures. Data consists of three biological and two technical replicates from separate cultures. <indicates survival was below the limit of detection of the assay (25 CFU/ml). Statistical analysis using linear regression (r^2^) and an interaction test (Chi^2^) was performed using the statistical program Stata 12. Bracket ∧ indicate a significant difference in concentration independent of PCR-ribotype (p<0.01), Bracket * indicates a significant difference between PCR-ribotypes independent of concentration (p<0.01) and Bracket ∧* indicates a significant difference between PCR-ribotypes in a concentration dependent manor (p<0.01).

CRAs are halogenic compounds widely used in disinfection regimes. The active ingredients in Actichlor® and Haztab® are sodium dichloroisocyanurate (NaDCC), Adipic acid and NaDCC respectively. The manufacturers' recommended working concentrations vary slightly for outbreaks and blood spills, but are conserved for general use (Table 1). The susceptibility of the 012, 017 and 027 ribotypes to chlorine releasing disinfectants revealed that at 5000 ppm survival was below the limit of detection of the assay in all ribotypes (Figure 2A and 2B), whereas at 1000 and 500 ppm, spores survived for all three ribotypes.) (Figure 2A and 2B). A Chi^2^ interaction test and a partial F-test revealed that although concentration and PCR-ribotype were linked, the efficacy of the disinfectants were concentration dependent, irrespective of PCR-ribotype for both Actichlor® and HazTab® (Tables 2 and 3). Overall, there were no significant differences between the efficacy of Actichlor® and HazTab® (Table 3).

**Table 2:** Chi^2^ and partial F-test *p*-vales.

Disinfectant	Chi^2^ *p*-value	Partial F-test *p*-value
Actichlor®	0.0004	0.0172*
Biocleanse®	0.0017	0.0000
Haztab®	0.0004	0.0164*
Hibiscrub®	n/a	0.0050∧;
Newgenn®	0.5131*	0.0000
Perasafe®	n/a	n/a
Prociene 40®	0.0610*	0.0000
Steri7®	0.0000	0.0026
Virkon®	0.0306*	0.0000

Chi^2^ *p*-value is the probability that the differences observed for each disinfectant are independent of strain and concentration, p<0.01 indicates that strain and concentration are both a factor in the efficacy of the disinfectant, whereas

*indicates there is no significant interaction between strain and concentration. A partial F-test was performed to determine whether there were significant differences between the three PCR-ribotypes (p<0.01). ∧ indicates the exception to the partial F-test, where the strain difference for Hibiscrub was tested using a three variant Chi^2^.

**Table 3:** Disinfectant efficacy estimated using coefficient of variance.

Disinfectant	coefficient of variance	*p*-value	Standard error
Perasafe®	−3.3024	0.000	0.296
Actichlor®	0	n/a	0.194
Haztab®	0.19	0.249 *	0.165
Biocleanse®	1.7004	0.000	0.358
Newgenn®	2.415	0.000	0.318
Steri7®	2.64	0.000	0.275
Virkon®	2.974	0.000	0.369
Prociene 40®	3.4749	0.000	0.267
Hibiscrub®	4.0972	0.000	0.292

A Linear regression was performed taking strain, concentration and disinfectant into consideration. Actichlor® was used as the reference and the output gave the coefficient of variance from the reference (p<0.01). This was then normalised to the reference to give the overall variance from Actichlor® The larger the negative coefficient of variance the higher efficacy of the disinfectant. The standard error and p-values are listed, where

*indicates no significant difference from the reference Actichlor® (*p*>0.01).

### Peroxygens

Peroxygens are oxidizing agents, two differently acting peroxygens were tested, Perasafe® and Virkon® (Table 1). The efficacy of these peroxygens was dependent on their mode of action. The survival rate for all PCR-ribotypes tested was below the limit of detection when treated with Perasafe®, which was consistent across all the concentrations tested (Figure 2C). Using a Linear regression model, factoring for strain (PCR-ribotype), concentration and disinfectant, we can estimate based on the coefficient of variance from the reference Actichlor®, that Perasafe® exhibited the lowest level of survival of all nine disinfectants under the test conditions (Table 3). Treatment with Virkon® revealed survival was PCR-ribotype dependent (p<0.01; Table 3), whereby survival of the 027 ribotype was significantly lower than the 012 and 017 ribotypes (Figure 2G).

### Quaternary ammonium compounds (QACs)

QAC's are cationic surfactants, four different QACs were tested, Biocleanse®, Newgenn®, Proceine-40® and Steri7® (Table 1). These surfactants were distributed into two categories, those whose efficacies were strain dependent irrespective of concentration (Newgenn® and Proceine-40®, Table 2) and those that were linked to both PCR-ribotype and concentration (Biocleanse® and Steri7®, Table 2). The PCR-ribotype 027 was more susceptible to treatment with Newgenn®, Proceine-40® and Steri7® (Figure 2D, 2E and 2H), whereas the PCR-ribotype 027 is more resistant to Biocleanse® (Table 2, Figure 2I). The recommended working concentrations of Biocleanse® and Steri7® are 5% and 100% respectively, however, the PCR-ribotype dependent differences indicate that for Biocleanse® the 027 ribotype is more susceptible to 5%, whereas the 017 ribotype is more susceptible to 10% and the 012 PCR-ribotype is more susceptible to 20% (Figure 2I). For Steri7® the 027 and 012 PCR-ribotypes are more susceptible to a concentration of 100%, whereas the PCR-ribotype 017 is more susceptible to a concentration of 40% (Figure 2H). A low level of survival of 012 vegetative cells was detected for Steri7® at 20%.

### Biguanides

The active ingredient in Hibiscrub is chlorhexidine gluconate (Table 1), which is widely used in hand wash. A three variant chi^2^ test was performed to determine the efficacy of Hibiscrub® (Figure 2F, table 3) the 027 PCR-ribotype was significantly more resistant to Hibiscrub® than the 012 PCR-ribotype and 017 PCR-ribotype (*p<*0.01) (Figure 2F).

## Discussion

The efficacy of disinfectants against the nosocomial pathogen *C. difficile* is central to infection control strategies, especially as colonization rates near infected individuals are as high as 58% [16]. Recent publications have indicated aerosolization of spores as well as environmental contamination contribute to dissemination of *C. difficile* [13]. The transmissibility and virulence of *C. difficile* is continually evolving, through ecological and environmental influences. The spores produced by *C. difficile* enhance transmission due to their ability to survive in the environment [18], [29], [30] and resists biocides [19], [20], [21]. Cross resistance has been observed between biocides and antibiotics [31], [32], [33], [34], which is enhanced by exposure to sub-inhibitory concentrations of biocide [35].

We show variation in sporulation rates of *C. difficile* PCR-ribotypes 012, 017 and 027. The toxin defective strain M68, an 017 PCR-ribotype, showed the highest sporulation frequency under these test conditions, with an average of 3.5 times the spore titre compared to 012 and 027 PCR-ribotypes. The high sporulation rate of the 017 PCR-ribotype, may have contributed to the transcontinental spread of the 017 PCR-ribotype, in spite of their lack of one of the major virulence factors, toxin A from this lineage. Limiting the transmission of *C. difficile* spores in healthcare settings is an important factor in infection control; however, even sporicidal disinfectants are relatively inactive against *C. difficile* spores, which are able to remain on various surfaces even after disinfection [36], [37]. Contaminated surfaces have been implicated as reservoirs for airborne transmission of spores, which can be aerosolized by disturbance of these contaminated environments [13]. The transmission of environmental spores and efficacy of disinfectants to prevent patient-to-patient transmission has recently been addressed using a murine model, in which oxidizing disinfectants had the most effective reduction in transmission efficiency of the 017 PCR-ribotype strain M68 [38]. This along with the data we present highlights the importance of disinfectant choice in limiting the spread of CDI.

In this study, with the exception of Perasafe®, the disinfectants fell into three categories, i) those whose efficacies were dependent on concentration, ii) those whose efficacy were dependent on PCR-ribotype and iii) those whose efficacies were dependent on both PCR-ribotype and concentration. The use of CRAs, peroxygen based compounds, QACs and biguanides is widespread in the hospital setting, with different biocides used for distinct applications, including; antiseptic, disinfectant or preservative treatments [39]. Biocide activity can be affected by several different factors, including; concentration, contact time, pH, temperature, organic matter, as well as the number and condition of the bacteria, such as vegetative cells, biofilms and spore [39]. Within our experimental system, the tests were performed on liquid cultures to enable direct comparisons to be made between different disinfectants. However, some of these disinfectants are surfactants, therefore the low level of activity of some of these compounds could be linked to the experimental methods used. The most effective biocides across all three PCR-ribotypes tested were the oxidizing agents, such as CRAs (Specifically Actichlor® and HazTab®) and peroxygens (specifically Perasafe®), which damage DNA, proteins and lipids [40]. It has been shown that oxidizing agents such as H_2_O_2_ interfere with the spore coat thus rendering the spore nonviable [38]. However, H_2_O_2_ has been shown to be less effective than other peroxygens [41]. There was a marked difference between the efficacies of two types of peroxygens tested. The peracetic acid containing peroxygen was active against all three PCR ribotypes, where the level of survival of *C. difficile* was below the limit of detection for the assay, indicating a 5–Log_10_ reduction in spore titre, whereas the 012, 017 and 027 PCR-ribotypes were less susceptible to the potassium peroxymonosulphate containing peroxygen, with less than a 1-Log_10_ decrease in spore titre at the recommended working concentration. However, there were PCR-ribotype dependent differences in the susceptibility to differing concentrations of Virkon®.

The CRAs Actichlor® and Haztab® showed a good efficacy at 5000 ppm and 1500 ppm, however, survival of spores was detected for all three PCR-ribotypes at lower concentrations, which is consistent with published data indicating that CRAs are only sporicidal at high concentrations [42].

Under the experimental methodology used in this study, the QACs were overall less effective against the three PCR-riboypes than the CRAs and the peroxygens, which may be linked to their use as mainly surfactants. However, interesting differences were observed between these disinfectants that were dependent on concentration and PCR-ribotype. The PCR ribotype 027 was more susceptible to the majority of the QACs (except Biocleanse®) and the peroxygen Virkon® than the 012 and 017 PCR-ribotypes. However, the 027 PCR-ribotype was more resistant to the widely used hand wash Hibiscrub® than the 012 and 017 PCR-ribotypes. All disinfectants exhibited effective inactivation of vegetative cells at the majority of concentrations tested, with a few exceptions at low concentrations.

The comparative efficacy of the nine disinfectants was assessed using a Linear regression model controlling for strain (PCR-ribotype), concentration and disinfectant, with Actichlor® as the reference. The coefficient of variance was used as an estimate for the relative efficacy of the disinfectants, with Perasafe® being the most effective disinfectant under the experimental conditions used.

The global spread of CDI has seen the increase in other PCR ribotypes such as 050 and 176 [43] which show a high level of evolutionary similarity to 027 PCR-ribotypes [7]. Genetic and evolutionary analysis of *C. difficile* revealed that it has a highly dynamic genome, comprising gene loss, gene gain, rearrangements and point mutations. The highly epidemic *C. difficile* lineages have evolved independently; therefore the hypervirulent 027, 017 and 012 clades are genetically distinct [7]. This may account for the differences observed in susceptibility to disinfectants. The independent evolution is apparent with the acquisition of fluoroquinolone resistance; one clade of the 027 lineage contains a mutation in *gyrB,* which encodes intrinsic resistance to fluoroquinolones. This genetic and evolutionary link may also be the case for resistance to disinfectants. The genome sequence of R20291 has revealed a number of efflux pumps and ABC transporters unique to this hypervirulent 027 PCR-ribotype [44], which may play a role in resistance to biocides.

## Materials and Methods

### Bacterial culture and media

*C. difficile* strains tested are as follows: R20291 a PCR-ribotype 027 from an outbreak at the Stoke Mandeville hospital, England, 2006, strain M68 a PCR-ribotype 017 from an outbreak in Dublin, Ireland 2006 and strain 630 a PCR-ribotype 012 isolated from a patient in Zurich, Switzerland 1982. These strains have been genetically and phenotypically characterized and are good representatives of their distinct lineages. Strains were stored at −80°C and cultured on C.C.E.Y Agar (Oxoid), supplemented with 4% egg yolk emulsion (Bioconnections), 1% defibrinated horse blood (TCS Biosciences), and cycloserine/cefoxitin antibiotic supplement (Bioconnections) for 1 to 2 days under anaerobic conditions, in a Modular Atmosphere Control System 500 (Don Whitney Scientific) at 37°C. All cultures were performed in duplicate. Primary liquid cultures were inoculated with three single colonies into 10 ml of pre-reduced Yeast Peptone (YP) broth (16 g/L Peptone, 8 g/L Yeast, 5 g/L NaCl_2_) with 0.2% (v/v) Tween 80 and incubated anaerobically for 24 hours on a shaking platform at 60 rpm. Secondary cultures were inoculated using 1/20 dilution of the primary cultures onto 40 ml of pre-reduced YP broth with 0.2% (v/v) Tween 80 and incubated anaerobically for 24 hours.

### Vegetative cells and spore counts

Vegetative cell counts were determined for all cultures, 1 ml of each duplicate culture was centrifuged at 8000 x g and washed with 1 ml of sterile phosphate buffered saline 1 x (PBS, Sigma), samples were centrifuged again and pellets were resuspended in 1 ml PBS, serially diluted in 1 x PBS and plated in duplicate onto blood agar base plates supplemented 7% (v/v) defibrinated horse blood (TCS) and 0.1% (w/v) taurocholate (Sigma). Bacterial counts were enumerated on plates after 24 hours and calculations were performed to give colony forming units per ml (CFU/ml).

Heat resistant spore counts: 1 ml of each duplicate culture was incubated at 56°C for 20 minutes to heat inactivate the vegetative cells. The heat resistant spores were then centrifuged and washed as outlined. Serial dilutions were performed in 1 x PBS and plated in duplicate onto blood agar base plates supplemented 7% (v/v) defibrinated horse blood (TCS Biosciences) and 0.1% (w/v) taurocholate (Sigma). Colony counts were enumerated on plates after 24 hours, and calculations were performed to give CFU/ml. Direct spore countes were also made from the liquid culture using a haemocytometer (Neubauer-ruled Bright Line counting chambers; Hausser Scientific) and a light microscope (Nikon) at 1000 x magnification.

### Disinfectant assays

The disinfectants used in the study and concentrations are described in Table 1. Disinfectant survival assays were performed by mixing 1 ml of each duplicate culture with 1 ml of disinfectant at the appropriate concentration to give the desired final concentration. These were incubated for 30 minutes before 1 ml of the samples was centrifuged, washed, serially diluted and plated as outlined above. CFU counts were plotted in Graphpad Prism (v4) as percentage survival compared to heat resistant spore counts, error bars are standard error of the mean (SEM). The limit of detection for the assay is 25 CFU/ml.

### Statistical analysis

For the comparison between total counts and spore counts, an unpaired two-tailed Students T-tests were performed in Graphpad Prism (v4), with a confidence interval of 95% (*p<*0.05). Analysis on the percentage spore production and the efficacy of the disinfectants was performed using Stata 12 statistical analysis program. Three questions were set to analyze the data i) is there a strain dependent sensitivity to the disinfectants? ii) if so, what is the most appropriate concentration to use? iii) which disinfectant has the greatest efficacy across all three PCR-ribotypes and concentrations? Hypotheses i and ii were answered using an interaction test (Chi^2^) performed on two Linear regression analyses (r^2^) using log_10_ percentage survival data: The linear regression analyses performed were a) the regression accounting for concentration (independent of strain) and b) the regression accounting for both strain (PCR-ribotype) and concentration. The Chi^2^ test to look for a relationship between strain and concentration was then performed on these two regression data sets (a and b), where a P<0.05 indicates that both strain (PCR-ribotype) and concentration are a factor in the efficacy of the disinfectant (Table 2). A partial F-test was performed to determine whether there was a significant difference between the PCR-ribotypes for each disinfectant at a confidence interval of 99% (p<0.001) (Table 2). When both the Chi^2^ interaction test (p<0.05) and partial F-test (P<0.01) gave significant difference between strain and concentration, the most appropriate concentration for a particular ribotype could be estimated. These estimates were calculated from the regression b (strain and concentration) using the coefficient of variance from the lowest concentration of a particular disinfectant. The lowest value or largest negative coefficient of variance from the control (lowest concentration) the more effective the disinfectant (Table S1), whereas the more positive the coefficient of variance the less effective the disinfectant under the test conditions (Table 3). Hypothesis iii was addressed using a linear regression controlling for strain, concentration and disinfectant, where the relative efficacy of the nine disinfectants was assessed using the coefficient of variance from Actichlor® the reference disinfectant. The largest negative coefficient of variance from the control (0) the more effective the disinfectant (Table 3), whereas the more positive the coefficient of variance the less effective the disinfectant under the test conditions (Table 3).

## Supporting Information

Table S1 **Concentration differences between PCR-ribotypes.** Where an interaction was detected by Linear regression taking strain and concentration into consideration. The lowest concentrationwas used as the reference for each disinfectant and the output gave the coefficient of variance from the reference (p<0.01). The larger the negative coefficient of variance the higher efficacy of the disinfectant.
(DOCX)
