# Supplementary material for: Hypervirulent Clostridium difficile PCR-Ribotypes Exhibit Resistance to Widely Used Disinfectants
Source: PLoS One. 2011 Oct 25;6(10):e25754. doi: 10.1371/journal.pone.0025754 (PMC3201945; doi:10.1371/journal.pone.0025754)
Supplement: Table S1 — Concentration differences between PCR-ribotypes. Where an interaction was detected by Linear regression taking strain and concentration into consideration. The lowest concentrationwas used as the reference for each disinfectant and the output gave the coefficient of variance from the reference (p<0.01). The larger the negative coefficient of variance the higher efficacy of the disinfectant. (DOCX) [file pone.0025754.s001.docx]

| Disinfectant | Strain | Concentration | Coefficient of Variance |
| --- | --- | --- | --- |
| Steri 7® | M68 | **40** | **-1.41467** |
|  |  | 80 | -0.7545 |
|  |  | 100 | -0.5645851 |
|  | 630 | 40 | 0.9738451 |
|  |  | 80 | -2.281844 |
|  |  | **100** | **-2.527909** |
|  | R20291 | 40 | 0.9177932 |
|  |  | 80 | 0.3420951 |
|  |  | **100** | **0.0348467** |
| Biocleanse® | M68 | 5 | -0.2652 |
|  |  | **10** | **-0.5151** |
|  |  | 20 | -0.1473 |
|  | 630 | 5 | -0.8276 |
|  |  | 10 | -2.0104 |
|  |  | **20** | **-2.247** |
|  | R20291 | **5** | **0.13703** |
|  |  | 10 | 0.6434 |
|  |  | 20 | 0.466 |
